# A New Strategy for Dietary Nutrition to Improve Intestinal Homeostasis in Diarrheal Irritable Bowel Syndrome: A Perspective on Intestinal Flora and Intestinal Epithelial Interaction

**DOI:** 10.3390/nu16183192

**Published:** 2024-09-21

**Authors:** Xinyu Wu, Yilong Cao, Yixiang Liu, Jie Zheng

**Affiliations:** 1 College of Ocean Food and Biological Engineering, Jimei University, Xiamen 361021, China; wxinyu2022@163.com (X.W.); 13860226510@163.com (Y.C.); 2 School of Chemistry and Chemical Engineering, Chongqing University, Chongqing 400044, China

**Keywords:** diarrheal irritable bowel syndrome, intestinal dysfunction, intestinal flora, functional populations of IECs, nutritional intervention, synergistic mechanism

## Abstract

Background and objectives: Although a reasonable diet is essential for promoting human health, precise nutritional regulation presents a challenge for different physiological conditions. Irritable Bowel Syndrome (IBS) is characterized by recurrent abdominal pain and abnormal bowel habits, and diarrheal IBS (IBS-D) is the most common, seriously affecting patients’ quality of life. Therefore, the implementation of precise nutritional interventions for IBS-D has become an urgent challenge in the fields of nutrition and food science. IBS-D intestinal homeostatic imbalance involves intestinal flora disorganization and impaired intestinal epithelial barrier function. A familiar interaction is evident between intestinal flora and intestinal epithelial cells (IECs), which together maintain intestinal homeostasis and health. Dietary patterns, such as the Mediterranean diet, have been shown to regulate gut flora, which in turn improves the body’s health by influencing the immune system, the hormonal system, and other metabolic pathways. Methods: This review summarized the relationship between intestinal flora, IECs, and IBS-D. It analyzed the mechanism behind IBS-D intestinal homeostatic imbalance by examining the interactions between intestinal flora and IECs, and proposed a precise dietary nutrient intervention strategy. Results and conclusion: This increases the understanding of the IBS-D-targeted regulation pathways and provides guidance for designing related nutritional intervention strategies.

## 1. Introduction

Irritable bowel syndrome (IBS) is a multifactorial functional gastrointestinal disorder characterized by recurrent episodes of abdominal pain and abnormal bowel habits. The global prevalence of IBS ranges from 4% to 15%, with a predominance of young and middle-aged people [1,2,3,4]. According to the Rome IV subtype classification criteria [1], IBS can be divided into IBS with diarrhea (IBS-D), IBS with constipation (IBS-C), IBS with mixed bowel habits (IBS-M), and undefined IBS (IBS-U). IBS-D accounts for 37–62% of IBS cases, representing the most common subtype in China [5]. The occurrence of IBS-D is closely related to diet, stress, intestinal barrier disruption, intestinal flora disruption, psychological effects, and genetics [6,7]. However, the pathophysiology and underlying molecular mechanisms of IBS-D remain unclear, limiting accurate diagnosis and rational treatment strategies. Although current research suggests that restrictive diets [8], medications [9,10] (e.g., rifaximin and 5-hydroxytryptamine (5-HT) receptor antagonists), Chinese medicine [11] (TCM prescriptions, acupuncture, and moxibustion), and fecal microbial transplantation [12,13] alleviate IBS-D symptoms, these approaches present challenges such as small application scope, limited efficacy, and adverse side effects. Therefore, analyzing the pathogenesis of IBS-D and exploring new intervention strategies are essential for addressing these issues.

With the development of modern medicine and molecular biology, studies have increasingly shown that intestinal epithelial cells (IECs) and intestinal flora do not exist in isolation, and that they interact to maintain intestinal homeostasis. Intestinal commensal bacteria can enhance the number of Paneth cells and induce specific antimicrobial peptides (AMP) expression, such as regenerating family member 3 gamma (RegIIIγ) [14]. Contrarily, IECs can express Toll-like receptors (TLRs) and nucleotide oligomerization domain (NOD)-like receptors (NLRs), enabling them to recognize and release antimicrobial substances to resist pathogenic bacterial invasion [15]. Intestinal homeostatic imbalance is a vital physiological feature of IBS-D. IBS-D patients experience impaired functional populations of IECs and altered intestinal flora compositions, abundances, and metabolite levels in different intestinal segments compared to healthy individuals [16,17]. Therefore, understanding the interaction between the intestinal epithelium and flora, and targeting them for precise intervention may identify new strategies for IBS-D treatment and intervention.

Diet and nutrition are crucial for treating many chronic gastrointestinal diseases. Studies have shown that dietary interventions are becoming increasingly important in the therapeutic aspects of IBS-D. Dietary patterns, such as traditional dietary advice (TDA), low FODMAP diets (LFD), and gluten-free diets (GFD), have been recommended for non-constipated IBS patients over the past decade [8]. However, the LFD and GFD interventions focus on limiting carbohydrate and fiber intake. Prolonged use of these approaches may result in nutritional deficiency and may even adversely impact the intestinal flora [18,19]. In addition, several clinical studies confirmed the efficacy of probiotic and prebiotic intervention in IBS [20]. Their mechanism of influence primarily involves the restoration of intestinal microecological balance and the improvement of intestinal immunity and barrier function by affecting the levels of short-chain fatty acids (SCFAs) [21,22,23]. Dietary nutrients based on intestinal-flora–intestinal-epithelial interactions to intervene in intestinal homeostasis in different physiological states, such as inflammatory bowel disease (IBD) and diabetes, are attracting increasing attention. For example, both VC and VD3 rely on the Notch-1 signaling pathway to regulate the expression of the intestinal tight junction (TJ) protein claudin-1, effectively repairing the intestinal mucosal barrier [24]. Yanai et al. [25] found that curcumin significantly affected ulcerative colitis (UC). It increased the abundance of intestinal butyric acid-producing bacteria and improved the intestinal mucosal permeability by inhibiting nuclear factor-kappa-B (NF-κB) activation and activating the aryl hydrocarbon receptor (AHR) signaling pathway [26,27]. Soluble dietary fibers (SDF) and Lycium barbarum polysaccharides (LBP) restore the ecological balance of intestinal flora by upregulating the abundance of Bifidobacteria, Lactobacillus, and intestinal SCFAs. These substances also stimulate intestinal immune cells to enhance intestinal barrier function by relying on the TLR-2 receptor to ameliorate chronic inflammation in diabetes mellitus [28]. Bifidobacteria, Lactobacillus, and SCFAs have been reported to promote mucus secretion, maintain mucus layer integrity, and inhibit intestinal inflammation [28]. Therefore, this paper innovatively proposes a new concept of comprehensive and targeted intervention for IBS-D. This involves a precise intervention strategy for dietary nutrition based on the concept of intestinal-flora–intestinal-epithelial interactions.

In recent years, relevant reviews have emphasized the effect of dietary nutrient intervention on intestinal flora and related diseases [29,30,31]. Therefore, this review explores IBS-D pathogenesis from the perspective of the interaction between the functional populations of IECs and intestinal flora. It focuses on essential micronutrients, probiotics and prebiotics, and polyphenolic nutrients. Additionally, it analyzes the nutritional properties of different dietary nutrients in restoring intestinal epithelial integrity and regulating the ecological balance of intestinal flora, as well as the potential application value of different nutritional combinations in improving intestinal homeostatic imbalance. The concept of intestinal-flora–intestinal-epithelium interaction proposed in this review should be considered a development direction for precise dietary nutrition intervention in IBS-D and other digestive tract diseases in the future, to provide a theoretical basis for scientific design of dietary patterns in the IBS-D population.

## 2. IBS-D Intestinal Homeostatic Imbalance and Physiological Properties

**Figure 1:**
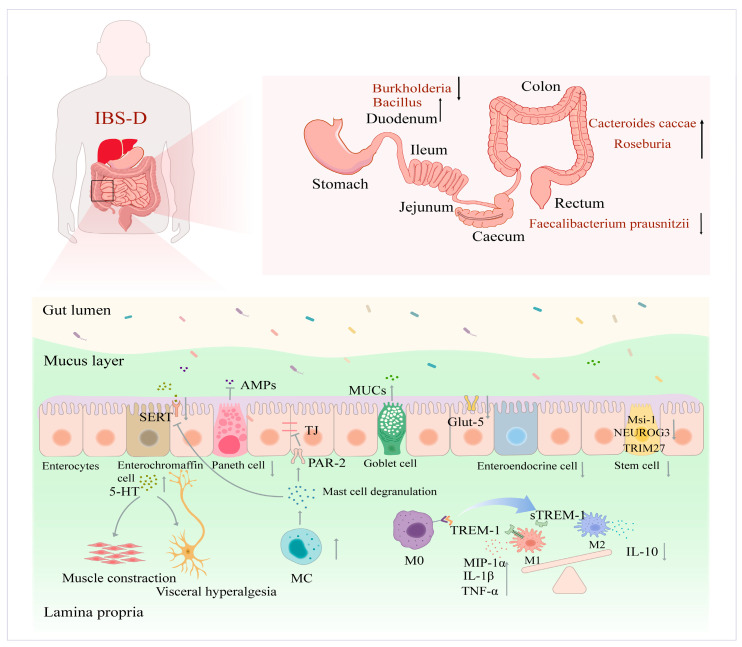
A schematic view of IBS-D pathogenesis. IBS-D: IBS with diarrhea, SERT: serotonin transporter, AMPs: antimicrobial peptides, MUCs: mucoproteins, TJ: tight junction, Glut-5: glucose transporter-5, Msi-1: Musashi 1, NEUROG3: Neurogenin 3, TRIM27: tripartite motif 27, PAR-2: protease-activated receptor 2, 5-HT: 5-hydroxytryptamine, MC: mast cell, M0: Macrophages 0, M1: Macrophages 1, M2: Macrophages 2, TREM-1: triggering receptor expressed on myeloid cells 1, sTREM-1: soluble triggering receptor expressed on myeloid cells 1, MIP-1α: mucosal inflammatory protein-1α, IL-1β: interleukin-1β, TNF-α: tumor necrosis factor-α, IL-10: interleukin-10, arrows: upward pointing arrows represent increases, downward pointing arrows represent increases, flat arrows represent inhibition.

IBS-D typically involves intestinal dysfunction with pathologic features that are not confined to a particular intestinal segment but extend throughout the intestinal tract, consequently making treatment more difficult. Evidence shows that IBS-D patients display significant homeostatic imbalance in the duodenum, jejunum, ileum, and colon compared to healthy controls (HC) [5,32,33]. This mainly reflects impaired functional populations of IECs and ecological imbalances in the intestinal flora (Figure 1). Therefore, intestinal homeostatic imbalance is an important physiological indicator of IBS-D development and can be targeted for related dietary nutrient intervention.

### 2.1. Impaired Functional Populations of IECs

The intestinal epithelium is a columnar monolayered epithelial structure. Epithelial cells can be divided into secretory cell populations, absorptive cell populations, intestinal stem cells (ISCs), and immune cells, according to their functions. These four distinct cell populations are interconnected and together define the main functions of intestinal epithelial tissues. IBS-D patients display impaired functional populations of IECs. Intervening in IBS-D by targeting different functional cell populations may be a meaningful idea for the future curing of IBS-D.

#### 2.1.1. Secretory Cell Populations

Secretory epithelial cell populations mainly include goblet cells, Paneth cells, and enteroendocrine cells (EECs), which are unevenly distributed in the intestine. Paneth cells are mainly in the small intestinal crypts, while EECs and goblet cells are distributed in the upper crypts and villi. These secretory cell populations secrete hormones, mucus, and antimicrobial peptides (AMPs), which defend against microorganisms, toxins, and antigens. Some studies have reported that IBS-D patients display increased small intestinal and colonic goblet cell activity and mucus secretion [34]. Paneth cells may affect visceral hypersensitivity in IBS-D patients. Visceral hypersensitivity refers to the reduction of the threshold of the patient’s internal organs for pain and other sensations. This increases visceral afferent nerve activation, while deactivating the corresponding brain regions, elevating the sensitivity of an organism to local stimuli [35]. Once the body organs are sensitive to intestinal motility, they can quickly recognize neuroexcitatory and temperature stimuli, leading to diarrhea, abdominal pain, and other symptoms. Therefore, visceral hypersensitivity has always been the focus of research in the pathogenesis of IBS-D. Lysozyme is an intestinal luminal AMP secreted by Paneth cells. Improving the lysozyme secretory function in Paneth cells effectively alleviates visceral hypersensitivity symptoms in IBS mice [36]. Studies have shown that fewer EECs at the duodenal site of IBS-D is closely related to visceral hypersensitivity, gastrointestinal motility disorders, and intestinal secretion abnormalities [37]. In addition, as the main EECs, enterochromaffin cells (ECs) secrete 90% of the 5-HT in the body, which is essential for regulating intestinal motility and sensory reflexes [38]. Research has shown higher 5-HT blood level in IBS-D patients [39,40,41]. Coates et al. [40] found significantly reduced serotonin transporter (SERT) expression in the intestinal mucosal epithelial cells of IBS-D patients. Therefore, it can be inferred that the decrease in the SERT expression obstructs 5-HT transmembrane transport, which increases the 5-HT level in the mucosal receptor-bounding state, and causes intestinal secretion, motility, and sensory reflex abnormalities. Therefore, the functional groups of secretory IECs influence IBS-D pathogenesis and can possibly be targeted for precise IBS-D intervention.

#### 2.1.2. Immune Cells

Immune cells influence immune defense, stabilization, and surveillance. Studies have shown significantly upregulated mucosal immune system activation in people with IBS-D, mainly in the form of increased immune cell infiltration and inflammatory cytokine expression. Significantly higher mast cell (MC) numbers and activation rates have also been reported in patients with IBS-D [42,43], which may induce and maintain low-grade inflammation. MCs can modulate epithelial barrier function and visceral sensitivity by releasing neuroactive mediators [44]. Martínez et al. [45] observed abnormal TJ protein expression in the jejunal mucosa of IBS-D people, which was positively correlated with the degree of MC activation. This was because the tryptase secreted by activated MCs stimulated the protease-activated receptor 2 (PAR-2) on the IECs to inhibit the expression of TJ proteins, which impaired intestinal epithelial barrier function [46]. MC degranulation also reportedly decreases SERT expression, which increases the 5-HT level in the mucosal receptor-binding state, exacerbating clinical symptoms in IBS-D patients [47].

Macrophages are important immune cells in the intestinal lamina propria. They can be divided into two polarized types, namely M1 (classically activated) and M2 (alternatively activated), according to the different macrophage phenotypes and the various cytokines they secrete. M1 is a pro-inflammatory macrophage, mainly activated by lipopolysaccharides (LPS) and interferon-γ (IFN-γ), which is capable of releasing interleukin-6 (IL-6), IL-1β, and tumor necrosis factor-α (TNF-α). M2 is an anti-inflammatory macrophage, which is mainly activated by IL-4, and releases IL-10 to inhibit inflammation [48]. Imbalanced macrophage polarization causes disproportionate pro-inflammatory and anti-inflammatory cytokine production, which leads to low-grade inflammation in IBS-D patients. Du et al. [49] demonstrated that serum-soluble triggering receptor expressed on myeloid cells 1 (sTREM-1) was significantly higher in IBS-D patients than in HC. sTREM-1 is the soluble form of the triggering receptor expressed on myeloid cells 1 (TREM-1), which is expressed in M1. Furthermore, IBS-D patients displayed higher intestinal mucosal inflammatory protein-1α (MIP-1α), TNF-α, and IL-1β levels, and lower IL-10 levels [50,51]. Therefore, macrophages in people with IBS-D may be polarized into M1, exacerbating their inflammatory response. In conclusion, the intervention of macrophage polarization into M2 may inhibit the inflammatory response and provide a new viewpoint and therapeutic means for curing IBS-D.

#### 2.1.3. ISCs and Absorptive Cell Populations

ISCs are presented at the base of the intestinal crypts and their main tasks are rapid renewal and repair of the IECs after injury [52]. IBS-D is closely related to the imbalance of ISC self-renewal and differentiation. Recently, Magdy et al. [53] revealed lower Musashi 1 (Msi-1) and Neurogenin 3 (NEUROG3) density in the duodenums of IBS-D patients than in HCs. Msi-1 and NEUROG3 represent markers of ISCs and intestinal endocrine progenitors, respectively. Therefore, the reduction in EECs may be related to abnormal ISC differentiation, which may crucially affect IBS-D development. In addition, Wang et al. [54] showed weak tripartite motif 27 (TRIM27) expression in IBS. TRIM27 knockdown induced spontaneous IBS-like symptoms. TRIM27 stabilizes β-catenin, which activates Wnt/β-catenin signaling, promotes ISCs renewal, and improves IBS symptoms. Therefore, precisely regulating ISC self-renewal and differentiation may be a novelty approach for IBS-D treatment.

The absorptive epithelial cell population is abundant on the villi, accounting for approximately 80% of IECs, and is the primary site responsible for nutrient absorption. Studies have found that more than 50% of pediatric IBS patients suffer from vitamin D (VD) deficiency [55], while micronutrient zinc deficiency has also been reported in patients with IBS-D [56]. These dietary nutrient deficiencies may be affected by the abnormal expression of relevant key transporter proteins on absorptive IECs. For example, the intestinal expression of the apical sodium-dependent bile acid transporter (ASBT), a transporter protein primarily responsible for bile acids (BAs) uptake in the terminal ileum, is reduced in patients with Crohn’s disease (CD), leading to BA malabsorption [57]. In addition, recent research has shown that fructose malabsorption is common in IBS-D patients, mainly due to reduced fructose absorption capacity resulting from insufficient glucose transporter (Glut-5) synthesis during high glucose intake [58,59]. Therefore, determining how intestinal absorptive epithelial cells regulate transporter protein expression and affect dietary nutrient absorption may be crucial for improving the symptoms of malnutrition in IBS-D patients in the future.

### 2.2. Ecological Imbalance of Intestinal Flora

Intestinal flora refers to the large number and variety of microbial colonies settled in the human intestinal tract. In normal physiological conditions, intestinal flora display a stable dynamic balance and, to a certain extent, can maintain the mucosal barrier, provide nutrients, regulate immune function, and resist pathogens [60,61]. Ecological dysbiosis refers to an imbalance in components or compositional quantity of intestinal flora, which has become an important pathophysiological mechanism of IBS.

The ecological imbalance of intestinal flora in IBS-D patients mainly manifests as reduced microbial diversity and stability. Meta-analysis has revealed that IBS-D patients display reduced gut microbial diversity and decreased probiotic bacterial abundance, such as Bifidobacterium and Lactobacillus, while the prominence of pathogenic bacteria increases [62]. As important microorganisms involved in intestinal immune homeostatic regulation, the decreased abundance of Bifidobacteria and Lactobacillus exacerbates abdominal pain symptoms in IBS-D patients [63,64]. However, the exact mechanism remains unclear. Studies have shown a decrease in the abundance of Burkholderia and a substantial increase in that of Bacillus in the duodenum. Decreased Burkholderia may affect intestinal probiotic colonization and indirectly promote intestinal inflammation [65]. Patients with IBS-D display abnormal BA metabolism and show increased BA synthesis or excretion [66]. Bacillus can inhibit fibroblast growth factor 19 (FGF19) expression, which negatively regulates BA synthesis in the intestine, and prompts the liver to synthesize and secrete more BAs via the gut-liver axis [65,66,67,68]. The intestinal flora of IBS-D patients also show a significant increase in the relative abundance of Pseudomonas, a bacterium that produces LPS, flagellin, and other toxins. This causes immune activation and intestinal mucosal barrier disruption, which exacerbates the clinical symptoms of IBS-D patients [69]. Studies have found that IBS-D patients have higher levels of Cacteroides caccae (a bacterium belonging to the Bacteroides group) and Roseburia (a bacterium belonging to the Clostridium group) in the colonic mucosa than the general population [70]. These two bacteria produce butyrate, which in turn facilitates 5-HT synthesis by distal colonic EECs [71]. A high 5-HT level is believed to be an important cause of exacerbated abdominal pain and diarrhea in IBS-D [72]. Furthermore, IBS-D patients reportedly show decreased Faecalibacterium prausnitzii abundance in the rectal area [73]. This bacterium is responsible for producing SCFAs [74] and anti-inflammatory proteins [75], which contribute to intestinal immunity and epithelial barrier formation. Therefore, a decline in Faecalibacterium prausnitzii may exacerbate intestinal inflammation and contribute to IBS-D development [65]. Gu et al. [76] found a substantial increase in the abundance of intestinal Fusobacterium nucleatum in IBS-D patients, which also exacerbated visceral hypersensitivity in rats with IBS-D.

## 3. The Interaction between the Functional Populations of IECs and Intestinal Flora

Functional populations of IECs and intestinal flora do not exist in isolation. They maintain a symbiotic, reciprocal relationship via close interactions. Exploring these interactions may provide guidance for therapeutic disease approaches. Recent studies have shown that highly diverse intestinal flora maintain a healthy intestinal epithelial barrier, while the functional populations of IECs can construct physical and chemical barriers against the invasion of pathogenic microorganisms.

### 3.1. Direct Interaction between Intestinal Flora and Epithelial Cells

**Figure 2:**
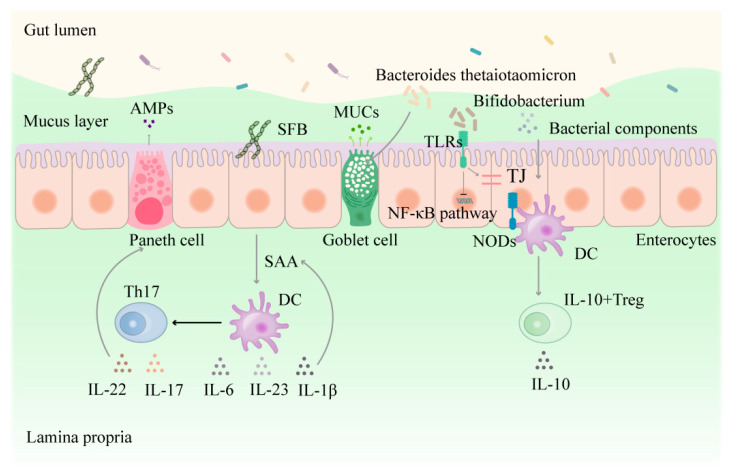
Interaction of intestinal flora and its constituents with intestinal epithelial cells. AMPs: antimicrobial peptides, MUCs: mucoproteins, SFB: segmented filamentous bacteria, TLRs: Toll-like receptors, TJ: tight junction, NF-κB: nuclear factor-kappa-B, NODs: nucleotide oligomerization domain, DC: dendritic cells, SAA: serum amyloid A, Th17: T helper 17 cell, Treg: regulatory T cells, IL-10: interleukin-10, IL-10 + Treg: Treg cells that secrete IL-10, IL-22: interleukin -22, IL-17: interleukin-17, IL-6: interleukin -6, IL-23: interleukin-23, IL-1β: interleukin-1β, arrows: facilitation, flat arrows: inhibition.

Intestinal flora are involved in maintaining intestinal epithelial homeostasis. Studies showed that the IEC proliferation rate in germ-free mice was lower than in conventional mice [77]. This difference suggests that intestinal flora can induce IEC proliferation. Segmented filamentous bacteria (SFB) can attach to ileal IECs to generate serum amyloid A (SAA), which leads to the production of the IL-1β, IL-6, and IL-23 cytokines by dendritic cells or monocytes. This stimulates T helper 17 (Th17) cell differentiation, providing further resistance to bacterial infection [78,79] (Figure 2). Laura et al. [80] indicated that Bacteroides thetaiotaomicron positively affected intestinal epithelial chemical barrier function by increasing goblet cell differentiation and regulating mucin-related gene expression. In addition, intestinal commensal Bifidobacteria are believed to have beneficial health effects by improving the intestinal barrier and attenuating mucosal inflammation [81]. Furthermore, components of intestinal flora, such as LPS and flagellin, can specifically bind to pattern recognition receptors (PRRs), such as NLRs and TLRs, expressed on IECs for immune surveillance. This regulates the signaling pathways involving NF-κB, mitogen-activated protein kinase (MAPK), and peroxisome proliferator-activated receptor γ (PPARγ) in IECs, and promotes IECs proliferation and the production of cytokines, antimicrobial proteins, and mucus [15]. Intestinal flora and its components are crucial for promoting IEC proliferation and differentiation, maintaining barrier function, and protecting the gastrointestinal tract from bacterial infection.

### 3.2. Interaction between Metabolites Derived from Intestinal Flora and Intestinal Epithelium

#### 3.2.1. SCFAs

**Figure 3:**
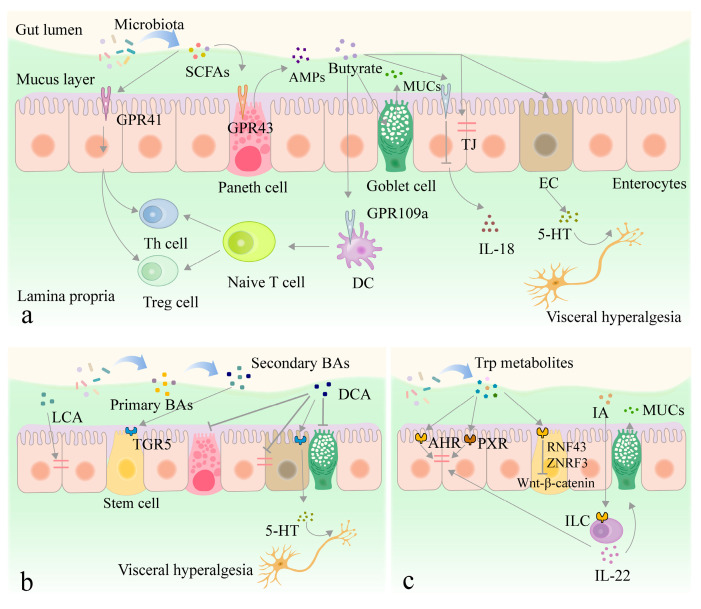
Metabolites of the intestinal flora maintain intestinal homeostasis ((**a**) SCFAs, (**b**) Second BAs, (**c**) Trp metabolites). SCFAs: short-chain fatty acids, AMPs: antimicrobial peptides, MUCs: mucoproteins, GPR41: G protein-coupled receptor 41, GPR43: G protein-coupled receptor 43, GPR109A: G protein-coupled receptor 109A, TJ: tight junction, EC: enterochromaffin cell, Th cell: T helper cell, Treg cell: regulatory T cell, DC: dendritic cell, IL-18: interleukin-18, 5-HT: 5-hydroxytryptamine, BAs: bile acids, LCA: lithocholic acid, DCA: deoxycholic acid, TGR5: G protein-coupled receptor5, Trp: Tryptophan, IA: indoleacrylic acid, AHR: aryl hydrocarbon receptor, PXR: pregnane X receptor, RNF43: ring finger protein 43, ZNRF3: zinc and ring finger 3,ILC: innate lymphoid cells, IL-22: interleukin-22. arrows: facilitation, flat arrows: inhibition.

The human diet contains a large amount of dietary fiber and indigestible carbohydrates, which can be fermented by intestinal flora to form SCFAs. Acetic acid, propionic acid, and butyric acid represent the most common SCFAs and are crucial for maintaining intestinal homeostasis by activating IECs to express G protein-coupled receptor 41 (GPR41), GPR43, and GPR109a [82,83,84,85] (Figure 3a). SCFAs also maintain intestinal homeostasis by promoting the expression of pancreatic islet regenerating family member 3 gamma (RegIIIγ) and beta-defensin via GPR43 in Paneth cells [86]. GPR41/43 receptor expression activates the extracellular regulated protein kinases 1/2 (ERK1/2) and p38 mitogen-activated protein kinase (p38MAPK) signaling pathways, inducing the production of chemokines and cytokines, thus effectively repelling pathogenic bacteria in the SCFAs [87]. Only butyric acid can activate the GPR109a receptor, the signaling of which promotes the anti-inflammatory properties of colonic macrophages and dendritic cells, and facilitates the differentiation of regulatory T cells (Treg) and IL-10-producing T cells [88]. Butyric acid also blocks the LPS/NF-κB pathway by activating the GPR109a receptor and promotes IL-18 secretion to regulate intestinal flora composition [89]. This is because IL-18 is closely associated with the production of mucins and AMPs. In addition, SCFAs enhance intestinal epithelial barrier function by promoting goblet cell differentiation and mucus production [90]. Paassen et al. [91] reported that butyric acid upregulated the expression of mucoprotein2 (MUC2), a mucin glycoprotein that is a major structural component of the mucus layer [92]. The mucus layer provides attachment sites for intestinal commensal bacteria and limits the binding of pathogens to IECs. In addition, SCFAs maintain intestinal epithelial barrier integrity by regulating TJ protein expression. Zheng et al. [93] found that IL-10 receptor α subunit (IL-10RA) expression was directly related to IEC barrier formation. This was because the upregulation of IL-10RA expression by butyric acid can inhibit claudin-2 expression by activating the signal transducer and activator of the transcription (Stat3) signaling pathway, increasing the physical barrier function. At the same time, it has been demonstrated that butyric acid enhances the interaction between specificity protein 1 (SP1) and the claudin-1 promoter. It also increases claudin-1 expression, which increases intestinal epithelial barrier function [94]. Finally, SCFAs act on ECs to alter gastrointestinal motility and secretion while regulating 5-HT secretion from ECs by affecting tryptophan hydroxylase 1 (TPH1) expression and activity [95]. Therefore, SCFAs may be important for intestinal 5-HT production and homeostasis in vivo [71,96]. In summary, SCFAs maintain intestinal homeostasis by regulating cytokine, mucin, TJ protein expression, and 5-HT secretion. Therefore, regulating SCFAs via dietary intervention may be a positive strategy for the future approach to GI disorders.

#### 3.2.2. Other Metabolites

(1) Secondary BAs can be divided into primary BAs containing cholic acid (CA) and chenodeoxycholic acid (CDCA), and secondary BAs containing deoxycholic acid (DCA) and lithocholic acid (LCA) [97,98]. Altered BA profiles modify intestinal epithelial permeability and influence barrier function by regulating the TJ protein expression. For example, LCA inhibits the TNF-α-induced reduction of zonula occludens (ZO) protein, occludin, and claudin-1 [99]. Li et al. [100] revealed that DCA disrupted intestinal epithelial barrier integrity, as evidenced by lower ZO-1 expression as well as the decline in goblet cells and Paneth cells (Figure 3b). Secondary BAs can act as signaling molecules to maintain intestinal homeostasis by interacting with intestinal epithelial-expressed BA activation receptors, such as G-protein-coupled BA receptor 1 (GPBAR1 or TGR5), farnesoid X receptor (FXR), vitamin D receptor (VDR), and pregnane X receptor (PXR) [101,102]. For example, the secondary BAs in the intestinal lumen promote ISC proliferation and differentiation via GPBAR1 after epithelial cell injury [103]. The secondary BAs also restore epithelial barrier integrity and intestinal flora diversity by binding to the FXR expressed by IECs [104]. LCA can act as a ligand for VDR to attenuate dextran sodium sulfate (DSS)-induced intestinal damage [105]. Alemi et al. [106] indicated that DCA stimulated the TGR5 on ECs to release 5-HT to promote colonic peristalsis, which was closely related to IBS-D pathogenesis.

(2) Tryptophan (Trp) metabolites indole (IND) and its derivatives, such as indole acetic acid (IAA), indole propionic acid (IPA), indole-3-ethanol (IEt), indoleacrylic acid (IA), and indole-3-carboxyaldehyde (I3A), are produced by the metabolism of Trp by intestinal flora capable of expressing tryptophanase. They can regulate the intestinal epithelial barrier by activating AHR and PXR [107] (Figure 3c). Various studies have shown that IND, IEt, IA, and IPA affect TJ protein expression by activating PXR [108,109]. Similarly, IEt, IPA, and I3A maintain intestinal epithelial integrity and attenuate DSS-induced colitis in dependent AHR [110]. Liu et al. [111] found that the IA produced by Parabacteroides distasonis promoted IL-22 secretion and alleviated the LPS-induced decrease in occludin, claudin-1, ZO-1, and MUC2 expression by activating AHR, positively affecting the restoration of intestinal chemical and physical barriers. Metidji et al. [112] indicated that the absence of AHR on the surfaces of IECs caused excessive ISC proliferation and abnormal differentiation. Trp metabolites restored intestinal epithelial barrier homeostasis and protected the ecological niche of stem cells by activating AHR. They restricted ISC proliferation by transcriptionally regulating ring finger protein 43 (RNF43) and zinc and ring finger 3 (ZNRF3) to inhibit Wnt-β-catenin signaling, effectively preventing the development of tumors. Scott et al. [113] showed that Trp metabolites reduced host actin regulatory protein expression by activating dopamine receptor D2 (DRD2). The protein aided hemorrhagic *E. coli* colonization of the intestine. Figure 3 summarizes the interactions of SCFAs, secondary BAs, and tryptophan metabolites with intestinal epithelial cells.

### 3.3. The Functional Populations of IECs and the Chemicals They Secrete Affect Intestinal Flora

The primary function of functional populations of IECs is to facilitate the absorption of nutrients, water, and electrolytes while preventing the invasion of harmful substances (e.g., bacteria and endotoxins). The functional populations of IECs and the chemicals they secrete can form physical and chemical barriers. Healthy gut barrier function plays a key role in regulating intestinal flora ecological balance and host defense.

**Figure 4:**
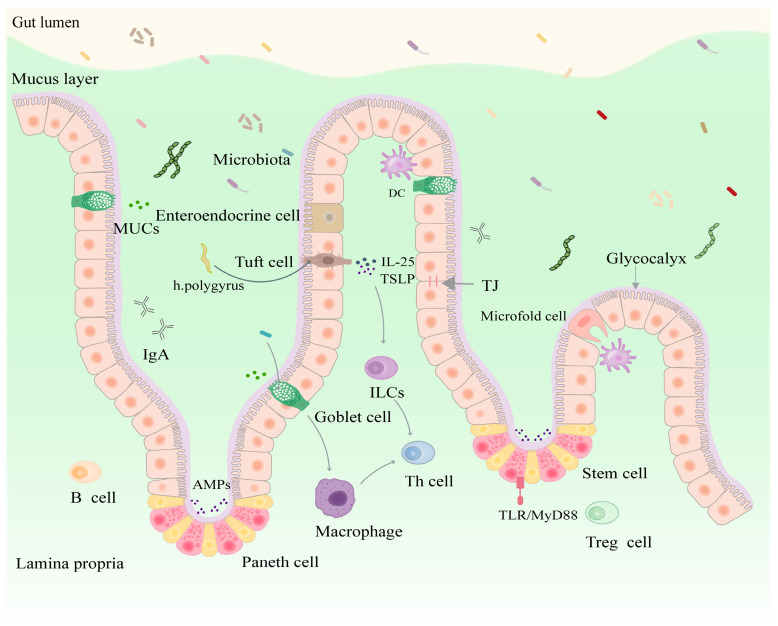
IECs affect the function of intestinal flora by forming physical and chemical barriers. DC: dendritic cell, MUCs: mucoproteins, IL-25: interleukin-25, TSLP: thymic stromal lymphopoietin, TJ: tight junction, IgA: immunoglobulin A, ILCs: innate lymphoid cells, AMPs: antimicrobial peptides, Th cell: T helper cell, TLR: Toll-like receptor, Treg cell: regulatory T cell, arrows: facilitation.

Physical gut barriers include various IECs, intercellular TJ proteins, and glycocalyx on the microvilli of absorptive IECs, while chemical barriers include AMPs, mucoproteins (MUCs), digestive juices, and other chemicals [114]. About 80% of IECs are enterocytes [115]. Goblet cells, the second largest group of IECs, promote lubrication and normal passage of intestinal contents by secreting mucus [116]. The mucus layer provides attachment sites for intestinal commensal microorganisms and serves as a growth substrate for beneficial microorganisms, contributing to their continued intestinal colonization [117]. MUC2, a major mucin component in mucus, binds to bacteria in the gut to keep them in the mucus layer. This prevents contact between bacteria and IECs and removes bacteria via intestinal motility [92]. Goblet cells can also deliver luminal antigens-to-antigen-presenting cells (APCs), eliciting an adaptive immune response, which has an important affect in combating bacteria [118]. Paneth cells are found exclusively in the small intestine and secrete AMP, such as C-type lectins and lysozyme [119,120]. Paneth cells induce AMP expression by activating the TLR/MyD88 signaling pathway sense gut bacteria. This ultimately limits bacterial penetration into host tissues and effectively helps maintain host–microbe homeostasis at the mucosal interface [121]. Recent studies have demonstrated that α-defensins enhance bacterial adhesion to epithelial surfaces to promote commensal Bacteroides colonization, preventing antibiotic-induced dysbiosis [122]. Tuft cells are chemosensory epithelial cells that produce IL-25 and thymic stromal lymphopoietin (TSLP), which induce a Th2 immune response to prevent helminth infection [123] (Figure 4). The neighboring IECs are basolaterally and apically connected by TJ proteins, which consist of transmembrane proteins (TP), junctional adhesion molecules (JAM), occludin, and claudins, which pass through the ZO occluden zone proteins to the actin cytoskeleton. These cellular connections selectively restrict the diffusion of small molecules, water, and ions; impede microbial invasion via the paracellular pathways; protect the body from infection; and are essential for maintaining physical barrier integrity [124,125]. The glycocalyx, a complex polysaccharide–protein structure covering epithelial cells, also acts as a barrier against invading bacteria on the surfaces of epithelial cells [126] (Figure 4). Studies have shown that IBS-D patients display lower jejunal glycocalyx levels, which disrupts the intestinal physical barrier and results in local immune activation [32].

In conclusion, the interactions between intestinal flora and functional populations of IECs are complex. Intestinal flora can directly or indirectly influence and regulate IECs, while IECs influence intestinal flora function via physical and chemical barrier formation. Despite a close connection between intestinal flora and IECs, the potential mechanism behind their interaction requires clarification to provide a theoretical basis for comprehensive intervention in gastrointestinal disorders, such as IBS-D, which exhibits a wide range of pathogenetic factors and complex pathogenetic mechanisms.

## 4. Dietary Nutrient Intervention in IBS-D

This paper identifies intestinal homeostatic imbalance as a key factor in IBS-D pathogenesis and examines interactions between the intestinal flora and functional populations of IECs. Next, it explores the possibility of dietary nutrient intervention for IBS-D, using essential micronutrients, probiotics and prebiotics, and polyphenols. These dietary nutrients are summarized in Table 1.

### 4.1. Essential Micronutrients

Vitamins and minerals are micronutrients essential for maintaining human life. Fat-soluble vitamins, such as vitamin A (VA), VD, and vitamin E (VE), water-soluble vitamins, such as vitamin B (VB), and minerals, such as zinc and Se, are vital for regulating intestinal barrier function. Their advantages in maintaining the intestinal barrier are described in detail below, providing a theoretical basis for the targeted design of dietary patterns for the IBS-D population.

#### 4.1.1. Vitamins

VA is an essential fat-soluble vitamin that is absorbed by IECs in carotenoids and retinyl esters, which are eventually metabolized to their active form, retinoic acid (RA). In vitro studies showed that VA regulates intestinal barrier function by altering TJ protein expression. VA-treated Caco-2 cell monolayers exhibit elevated transepithelial electrical resistance (TEER) [127]. VA stimulates intestinal development in weaned piglets by inducing ISC differentiation and increasing the number of EECs in the villi [128]. Lisa et al. [129] demonstrated that RA promotes the secretion of IL-22 by immune cells. IL-22 can induce the AMPs production by Paneth cells, which inhibits DSS-induced colitis. In addition, during intestinal infection, VA stimulates IL-18 secretion by IECs, which promotes the shedding of infected epithelial cells and encourages immune cells to produce IFN-γ for further pathogen clearance [131].

VD is a fat-soluble vitamin essential in the human body. Various types of VD exist, with the most significant ones being vitamin D2 (VD2, ergocalciferol) and vitamin D3 (VD3, cholecalciferol). VD2 is mainly derived from plant sources, while VD3 is obtained from animal sources [164]. Studies have shown that VD increases quality of life of IBS patients [165,166,167,168]. This may be because VD helps to maintain intestinal epithelial barrier function. VD3 reportedly has a repairing effect on the intestinal TJ damage induced by DSS [132], TNF-α [133], and alcohol [134]. A study by Zhou [135] showed that VD induced MUC2 and goblet cell-associated gene expression, which was closely related to mucus layer integrity. In addition, the expression level of resistin-like molecule β (RELMβ), a hormone secreted by goblet cells, was significantly elevated in VD-fed mice, which helped enhance the colonic barrier and regulate colitis [135,169]. Dong et al. [134] used cell and animal models to demonstrate that VD3 significantly reduced the MyD88 expression and ZO release associated with TJ protein damage, which addressed the issue of increased intestinal barrier permeability.

B vitamins help maintain the integrity of the intestinal barrier. VB3 reduces epithelial cell apoptosis and promotes epithelial cell renewal by activating the D prostanoid receptor 1 (DP1) in colonic epithelial cells [137]. Reduced VB6 intake exacerbates IBS symptoms in a cross-sectional study [138], possibly because lower VB6 consumption disrupted pro- and anti-inflammatory cytokine homeostasis [139]. Research showed that VB12 upregulated the expression of trefoil factor peptides 1/2/3 (TFF1/2/3), mucin-encoding genes (MUC13), and TJ proteins (claudin-2 and claudin-18), which were closely related to mucin secretion, revealing the potential role of VB12 in regulating intestinal barrier function. In addition, VE has a protective effect on intestinal health [136]. Studies showed that α and γ tocopherols inhibited colitis-induced occludin loss and reduced the plasma levels of LPS-binding proteins, which protected intestinal barrier integrity, as shown by a DSS-induced colitis mouse model.

#### 4.1.2. Minerals

Zinc is an essential mineral that is vital for maintaining intestinal barrier function [170]. Studies showed that zinc deficiency disrupts intercellular TJ proteins and increases intestinal permeability [171]. Shao et al. [140] demonstrated in vitro that zinc sulfate improved intestinal barrier function by promoting ZO-1 expression via the PI3K/Akt/mTOR signaling pathway using Caco-2 cells. In addition, high ZnO levels of 2000–3000 mg/kg have been widely used to prevent diarrhea symptoms in weaned piglets. One way was to reduce intestinal permeability by increasing occludin and ZO-1 expression [141]. In the chemical barrier, zinc deficiency affects mucin secretion while altering the critical structure of O-glycans and increasing shorter O-glycan production. This alters the O-glycan pattern and decreases intestinal mucus layer stability [142]. Additionally, zinc regulates Paneth cell resistance to pathogenic bacteria by maintaining lysozyme stability [172]. In summary, zinc can support the physical barrier by repairing the TJs between the IEC. It can also strengthen the chemical barrier function by maintaining mucosal layer stability and promoting AMP production. Therefore, zinc supplementation can potentially improve IBS-D symptoms.

Se is an essential micronutrient with antioxidant, anticancer, and antiviral properties. Dietary supplementation with Se nanoparticles (SeNPs) upregulates MUC2 and RegIIIγ expression in the jejunum, suggesting that biogenic SeNPs effectively mitigate diquat-induced intestinal barrier dysfunction [143]. Li et al. [144] prepared selenylation α-D-1,6-glucan (sCPA), an organic Se compound. This compound increased occludin and claudin-1 expression, ameliorated goblet cell damage in mice with colitis, and promoted mucus secretion, allowing it to maintain intestinal physical and chemical barrier integrity. Studies have revealed the ability of Se to convert M1-type macrophages into M2-type macrophages [145]. Other micronutrients can also be involved in maintaining barrier integrity. Dietary copper altered intestinal barrier function by modulating the intestinal hypoxia-inducible factor-1α (HIF-1α) signaling pathway and oxidative stress in alcoholic liver disease [146]. HIF-1α has been implicated in intestinal barrier integrity and inflammation, while HIF-1α deficiency increases epithelial permeability [173]. In conclusion, supplementation with essential micronutrients may alleviate IBS-D development by maintaining intestinal epithelial barrier function.

### 4.2. Probiotics and Prebiotics

#### 4.2.1. Probiotics

Probiotics are a group of living microorganisms that regulate the ecological balance of intestinal flora or mucosal immunity by colonizing the intestinal tract and conferring health benefits to the host [174]. Recognized intestinal probiotics include Bifidobacterium, Lactobacillus, Bacillus, and Streptococcus [175]. Sun et al. [147] evaluated the therapeutic efficacy and safety of Clostridium butyricum (CB) in IBS-D patients. The results showed that IBS-D patients with symptomatic relief displayed reduced fecal Clostridium counts. This study suggests that CB reduces overall IBS-D symptoms by decreasing Clostridium counts and further improving abnormal BA metabolism. In addition, using probiotics to modulate the intestinal flora can increase the number of bacteria producing SCFAs. Clinical trials demonstrated that oral administration of the L. plantarum CCFM8610 probiotic strain significantly reduced IBS-SSS and BS-QOL scores and restored intestinal flora diversity. Furthermore, it decreased the relative abundance of Methanobacterium, which is positively correlated with the degree of bloating, and increased the relative abundance of Anaerostipes, Anaerotruncus, Bifidobacterium, Butyricimonas, and Odoribacter, which are capable of producing butyric acid [148]. Notably, combining multiple probiotic strains can synergistically improve IBS-D symptoms. This was demonstrated in a study where multi-strain treatment with 14 probiotic strains significantly improved patients’ IBS-D symptoms and quality of life [149]. Moreover, probiotic supplementation synergistically promotes VD absorption. Cheng et al. [150] found that Lactobacillus rhamnosus LGG upregulated VD3 transporter protein (Cluster Determinant 36 (CD36) and Niemann-Pick C1-Like1 (NPC1L1)) expression, which promoted intestinal VD3 absorption and improved VD deficiency in IBS-D patients.

#### 4.2.2. Prebiotics

Although prebiotics cannot be digested in the stomach or small intestine, they can be selectively fermented in the colon by specific resident intestinal flora, thereby stimulating the growth of probiotics in the gut [152]. Fructo-oligosaccharides (FOS), SDF, and galacto-oligosaccharides (GOS) display the highest prebiotic effect and are mainly found in grains, vegetables, and legumes [176]. Probiotics are effective in preventing and treating a wide range of abnormal gut flora and inflammation-related diseases. For example, SDF reversed the abnormally elevated Bacteroidetes/Firmicutes (B/F) ratio and increased the concentrations of acetic acid and butyric acid, inhibiting the abnormal activation of intestinal glial cells to reduce colonic inflammation in mice with type 2 diabetes mellitus (T2DM) [151]. SDF and oligofructose stimulate the growth of Bifidobacteria, which is negatively correlated with Bifidobacteria concentration in vivo [177]. Therefore, it is logical to use prebiotics to regulate intestinal flora imbalance and reduce IBS-D symptoms. Chen et al. [152] used a novel PB consisting of FOS, GOS, SDF, and anthocyanins to intervene in IBS mice. The results indicated that the PB intervention substantially improved the compositional changes in the intestinal flora and intestinal epithelial barrier by regulating TJ expression, which reduced the IBS symptoms of the mice. This paper demonstrates that the synergistic effect of multiple prebiotics improves gastrointestinal symptoms. However, minimal studies are available regarding the ability of prebiotics to improve the health of people with IBS. For example, Wilson et al. [178] showed that prebiotics failed to improve symptoms in IBS patients. On the contrary, another clinical trial demonstrated that when patients were given β-galacto-oligosaccharides(β-GOS), symptoms such as flatulence and abdominal pain were effectively improved, increased levels of prebiotics, such as Bifidobacterium [153]. Their impact is controversial and influenced by prebiotic type and dosage, as well as the IBS subtype.

Overall, using specific probiotic and prebiotic supplementation to restore ecological balance of the intestinal flora and promote formation of beneficial metabolites may provide new strategies for managing IBS-D. However, current research is limited by probiotic and prebiotic selection and dosage. Therefore, selecting probiotics and prebiotics for IBS treatment must be based on a comprehensive consideration of the cause of the disease, the disease phenotype, the dosage, and the duration to maximize their effect.

### 4.3. Polyphenols

Polyphenols, a class of structurally diverse secondary metabolites found in plants, have attracted great attention due to their anti-inflammatory properties [179]. Resveratrol, a plant-derived polyphenol, has been demonstrated in in vitro cellular experiments to ameliorate inflammation by downregulating the overexpression of pro-inflammatory cytokines through modulation of TLRs and NF-κB signaling pathways [154]. Meanwhile, resveratrol can also attenuate inflammation in colitis mice by restoring the diversity of intestinal flora, rebalancing probiotics and pathogens [155]. Curcumin is a dietary polyphenol extracted from turmeric rhizomes. Li et al. [156] revealed that curcumin activated the PPARγ in macrophages to induce M2 polarization and further secrete IL-10 to promote its anti-inflammatory function. Quercetin is a flavonoid showing anti-inflammatory bioactivity. A study showed that 30 mg/kg quercetin effectively reduced colitis symptoms in mice, inhibited IL-17, TNF-α, and IL-6 production, and promoted IL-10 production [157]. Apple peel polyphenols (APP) have excellent anti-inflammatory properties. He et al. [158] extracted APP from apple peel powder, according to the bioactivity-guided fractionation method, and found that it downregulated the expression of TLR4 and NF-κB proteins at both transcriptional and translational levels, which protects the intestinal tract against harmful bacterial invasion. Moreover, APP promotes Bifidobacteria and Lactobacilli proliferation, increases the SCFA content, and displays potential prebiotic properties [180]. Chlorogenic acid (CGA), a dietary phenolic acid, blocks the NF-κB pathway by down-regulating CD14 and p65 expression, preventing the entry of phosphorylated p65 into the nucleus, and inhibiting TNF-α, IL-1β, and IL-6 secretion. This demonstrated the anti-inflammatory effect of CGA [159]. Research has shown that caffeic acid, a CGA metabolite, regulates the intestinal flora in mice with DSS-induced colitis. Kurarinone (KAR) is a flavonoid derived from Sophora flavescens. Xu et al. [160] demonstrated that KAR inhibited macrophage activation and pro-inflammatory cytokine expression. KAR significantly upregulated the expression of AHR in macrophages, further promoting IL-10 secretion, through establishing a 2,4,6-trinitrobenzenesulfonic acid (TNBS)-induced mouse model of IBS; meanwhile, clinical experiments showed that AHR expression in macrophages of IBS patients was negatively correlated with IBS severity, therefore, KAR may be an important dietary polyphenol for future intervention in the development of IBS. 

Recent studies have explored the possibility that polyphenols promote the growth of beneficial flora and described their effect on intestinal flora [181]. For example, Tomas et al. [182] revealed that polyphenols upregulated some intestinal bacteria abundance such as Akkermansia, Faecalibacterium, and Roseburi, while Faecalibacterium spp and Roseburi spp represented the most important and abundant bacteria involved in butyrate production. Berries are rich in phenolics such as phenolic acids, flavonols, and anthocyanins, which reportedly reduce the Bacteroidetes/Firmicutes ratios (B/F) and upregulate the abundance of Bifidobacterium, Lactobacillus, and Akkermansia. This suggests that berry polyphenols positively modulate the composition of intestinal flora [183]. Anthocyanins are water-soluble pigments and flavonoid compounds. Besides promoting SCFA production, anthocyanin-rich diets can restore bacterial community diversity and rebalance probiotics and pathogens to maintain intestinal homeostasis [162,184]. Similarly, oral resveratrol and quercetin administration can restore the richness, abundance, and homogeneity of gut microbes [155,157]. However, the biological effects of resveratrol in vivo are mostly characterized by low bioavailability, which is a limitation of its use as a drug or dietary supplement. Multi-target studies should be conducted in the future to improve the bioavailability of resveratrol [185]. In addition, dietary polyphenols can support intestinal epithelial barrier function. Resveratrol, quercetin, ellagic acid, and anthocyanins help repair and maintain intestinal chemical and physical barriers by increasing mucin expression and secretion and promoting inter-epithelial TJ protein expression [162,186,187,188].

### 4.4. The Synergistic Ability of Different Dietary Nutrients to Improve Intestinal Homeostasis

Combining dietary nutrients with different functions to regulate and improve intestinal homeostasis forms the basis for developing targeted dietary patterns for those with IBS-D. For example, vitamins, probiotics and prebiotics, and polyphenols can influence 5-HT synthesis and secretion, which can improve abdominal pain symptoms in IBS-D. Intestinal flora disorders can affect 5-HT synthesis in ECs, leading to chronic abdominal pain. VD may indirectly affect 5-HT levels by improving intestinal flora, consequently alleviating IBS-D symptoms [189,190]. A recent study indicated that L. plantarum AR495 effectively inhibited intestinal motility by restoring the luminal 5-HT levels, which restricted abnormal EC cell proliferation and TPH1 expression [191]. This study provides additional evidence that probiotic intervention IBS-D and other diarrheal diseases. Qin et al. [192] found that quercetin intervention reduced EC cell density and TPH expression in post-infectious IBS (PI-IBS) rats. Both pathways reduced colonic 5-HT bioavailability, further alleviating visceral allergy symptoms.

A VD and curcumin combination displayed a therapeutic and preventive ability in an acetic acid-induced model of acute UC, significantly reducing inflammation and restoring the normal tissue structure of the colon [193]. Studies suggest that VD and probiotic co-supplementation synergistically modulate intestinal microbiota and metabolomics. The use of probiotics increases intestinal VD absorption [194]. Research showed that combining VD3 and the p40-secreting probiotic Lactobacillus rhamnosus GG (LGG) synergistically ameliorated colitis in mice by increasing the expression of VDR in colonic and promoting colonic IECs proliferation. Studies showed that combining probiotics and polyphenols improves the intestinal barrier and regulates intestinal flora [195,196].

In summary, combining essential micronutrients, probiotics and prebiotics, and polyphenols for multi-targeted, synergistic intervention in IBS-D to restore epithelial integrity, balance ecological flora, mitigate inflammation, and 5-HT may provide a new strategy for future IBS-D treatment.

## 5. Results and Outlook

IBS-D with recurrent symptoms is a functional GI disorder and lacks clear evidence of organic GI tract pathology. To date, superficial understanding of the IBS-D pathomechanisms has limited effective therapeutic approaches. No specific drugs are available for comprehensive IBS-D intervention, restricting effective treatment. Recent research in molecular biology has shown that the intestinal homeostatic imbalance caused by impaired functional populations of IECs, and ecological imbalance of intestinal flora are key factors contributing to the development of IBS-D. In addition, a complex interaction is evident between the functional populations of IECs and the intestinal flora. This interaction involves the transmission of signals from the intestinal flora to the intestinal epithelium, either through direct or indirect means via its metabolites. Similarly, the functional populations of IECs may also secrete cytokines that affect the function of intestinal flora. Understanding these intricate interactions is essential for maintaining intestinal homeostasis.

Fortunately, studies are increasingly acknowledging the significant impact of nutrition on IBS-D intervention. However, nutrition science has traditionally focused on using a single nutrient to study its effects on certain diseases. Considering the complex pathomechanisms of IBS-D, single-nutrient intervention cannot holistically improve the symptoms of IBS-D patients. Therefore, the synergistic intervention of multiple dietary nutrients in IBS-D represents the future trend for treating IBS-D. Understanding the complex mechanism of nutritional synergies is highly significant for formulating dietary strategies to maintain human health and improve illness.

However, the pathogenesis of IBS-D is complex, severely affecting patients both physically and psychologically and placing a significant financial burden on the healthcare system. At the same time, the interaction between the functional populations of IECs and intestinal flora and IBS-D pathogenesis requires clarification. Minimal studies are available on using multiple nutrients for synergistic IBS-D intervention. Therefore, future research should explore the effect of intestinal flora and functional populations of IECs on IBS-D development and investigate the synergistic mechanism behind multiple dietary nutrients to realize a multi-target, multi-pathway, multi-nutritional strategy for IBS-D intervention. Designing nutritional compositions may become an effective strategy for future dietary supplementation and optimal dietary regimen for future interventions for IBS-D and other gastrointestinal disorders.

## Figures and Tables

**Table 1:** Possible dietary nutrient intervention pathways for IBS-D.

Nutrient Type	Component	Subjects	Main Findings and Markers	Effects	References
Essential micronutrients	VA	In vitro	TEER ↑, ZO-1 ↑, occludin ↑, and claudins ↑.	Regulates intestinal barrier function.	[127]
		Piglets	Induction of ISC differentiation, number of EECs ↑.	Promots intestinal development in weaned piglets.	[128]
		Mice	IL-22 ↑ and AMP ↑.	Inhibits DSS-induced colitis.	[129,130]
		Mice	IL-18 ↑ and IFN-γ ↑.	Limits pathogen invasion and activated immune cells to facilitate pathogen clearance during the early stages of infection.	[131]
	VD	In vitro and mice	TEER ↑, LPS ↑, and TJ ↑.	Repaires intestinal barrier damage.	[132]
		In vitro	NF-κB/MLCK-P-MLC ↓ and VDR ↑.		[133]
		In vitro and mice	TEER ↑, MyD88 ↓, and zonulin ↓.	Increases intestinal permeability.	[134]
		Mice	MUC2 ↑, goblet cell count ↑, and RELMβ ↑.	Enhances colonic barrier function and modulates colitis.	[135]
	VE	Mice	LPS ↓ and occludin ↑.	Maintains barrier integrity.	[136]
	VB_3_	Mice	Activated DP1, reduced apoptosis, and promoted renewal of IECs.		[137]
	VB_6_	Humans	Lower VB_6_ intake disrupts the balance between pro- and anti-inflammatory cytokines.	Severe IBS symptoms associated with lower VB_6_ intake.	[138]
	VB_12_	In vitro	TFF1/2/3 ↑, MUC13 ↑, and claudin2/18 ↑.	Reveals critical impact of VB_12_ in regulating cellular transcription, metabolism, and epigenetic programs.	[139]
	Zinc	In vitro	Activates the PI3K/Akt/mTOR signaling pathway, ZO-1 ↑.	Zinc maintains intestinal barrier function, regulated cell signaling and protein kinase activity.	[140]
		Piglets	occludin ↑, ZO-1 ↑.	Improves performance and reduces intestinal permeability in weaned piglets.	[141]
		In vitro	Zinc deficiency affected mucin secretion, structure and stability of the mucus layer.	Zinc is important for formation and maintenance of the physical intestinal epithelial barrier.	[142]
	Selenium	Mice	MUC2 ↑ and Reg IIIγ ↑.	Repaires intestinal barrier damage.	[143]
		Mice	occludin ↑ and claudin-1 ↑. Ameliorates goblet cell injury.	Maintaines the integrity of the physical and chemical intestinal barriers.	[144]
		In vitro	M2 macrophages ↑.	Optimal Se status is essential for M2 macrophage activation, attenuating pro-inflammatory mediator expression.	[145]
	Copper	In vitro, mice	Activates the HIF-1α signaling pathway. occludin ↑.	Copper homeostasis is essential for maintaining intestinal barrier integrity.	[146]
Probiotics	*Clostridium butyricum* (CB)	Humans	*Clostridium difficile* ↓.	Improves clinical symptoms.	[147]
	*Lactobacillus plantarum* (*L. plantarum*) CCFM8610	Humans	IBS-SSS ↓, IBS-QOL ↓, and the relative abundance of butyric acid-producing strains ↑.	Significant relief of clinical symptoms and intestinal dysbiosis in IBS-D people.	[148]
	Multi-strain probiotic preparation (14 probiotic strains)	Humans	Significantly improve the severity of abdominal pain ↓, the number of bowel movements per day, IBS-SSS ↓, and IBS-QOL ↓.	Multi-strain probiotics are associated with significant symptom improvement in IBS-D patients and are well tolerated.	[149]
	Lactobacillus rhamnosus GG	Mice	CD36 ↑, NPC1L1 ↑, SR-B1 ↓.	Lactobacillus rhamnosus GG culture supernatant promotes intestinal absorption VD by affecting protein levels of VD transporters.	[150]
Prebiotic	SDF	In vitro, mice	*Bacteroidetes*/*Firmicutes* ↑, IL-1β ↓, IL-6 ↓, TNF-α ↓, butyric acid ↑, and acetic acid ↑.	Restores intestinal dysbiosis and normalized the SCFA concentration in the colon.	[151]
	New prebiotic blend (PB)	In vitro, mice	occludin ↑, activates the PPARγ signaling pathway and regulates intestinal flora.	Significantly reduces IBS symptoms and regulates the gut flora.	[152]
	β-GOS	Humans	Bifidobacterium ↑, flatulence ↓, abdominal pain ↓.	Effective relief of IBS clinical symptoms.	[153]
Polyphenol	Resveratrol	In vitro	M1 macrophages ↓, IL-1β ↓, IL-6 ↓, TNF-α ↓	It has a significant anti-inflammatory effect and prevents disease progression via the TLR4/NF-κB signaling pathway.	[154]
		Mice	*Akkermansia* ↓, *Dorea* ↓, *Sutterella* ↓, *Bilophila* ↓, and *Bifidobacterium* ↑.	Alleviates gut microbiota dysbiosis.	[155]
	Curcumin	Rats	PPAR-γ activity ↑, IL-6 ↓, TNF-α ↓, and NO ↓.	Anti-inflammatory and antioxidative.	[156]
	Quercetin	Mice	IL-1β ↓, IL-17 ↓, IL-6 ↓, TNF-α ↓, *Bacteroides* ↑, *Bifidobacterium* ↑, *Lactobacillus* ↑, and *Clostridia* ↑, *Fusobacterium* ↓, and *Enterococcus* ↓.	Dietary quercetin directly stimulates the immune system, reduces inflammation, and restores gut flora balance.	[157]
	Apple peel polyphenol	Mice	TLR4 ↓, NF-κB ↓, and pro-inflammatory factors ↓.	Relieves intestinal inflammation.	[158]
	Chlorogenic acid	In vitro, mice	IL-1β ↓, IL-6 ↓, TNF-α ↓, and CD14 ↓. Blocks the NF-κB signaling pathway.	Elucidates the mechanism by which CGA inhibits inflammation and protects intestinal barrier function.	[159]
	Kurarinone	Humans, mice	Macrophage activation ↓, pro-inflammatory cytokine expression ↓, anti-inflammatory cytokines, such as IL-10 ↑.	Uncovers the mechanism of how KAR regulates macrophage function.	[160]
	Caffeic acid	Mice	IL1β, IL-6, TNF-α mRNA, protein levels ↓, IL-10 mRNA and protein levels ↓, ROS ↓, LPS ↓, and butyric acid ↑.	Alleviates DSS-induced colitis and improves defense against oxidative stress and inflammatory response.	[161]
	Anthocyanin	Mice	Chorion length, number of goblet cells, SCFAs, *Lachnospiraceae*, *Bacteroidaceae*, *Ruminococcaceae* ↑, and *Shigella* ↓.	Improves intestinal barrier function impairment and intestinal flora dysbiosis.	[162]
	Ellagic acid	Piglets	TJ protein ↑, DAO ↓, and goblet cell count ↑. Improves intestinal damage.	Restores intestinal barrier integrity.	[163]

VA: vitamin A, VD: vitamin D, VE: vitamin E, VB_3_: vitamin B3, VB_6_: vitamin B6, VB_12_: vitamin B12, SDF: soluble dietary fibers, β-GOS: β-galacto-oligosaccharides, TEER: transepithelial electrical resistance, Zo-1: zona occludens 1, IL-22: interleukin-22, IL-18: interleukin-18, INF-γ: interferon-γ, LPS: lipopolysaccharides, TJ: tight junction, NF-κβ: nuclear factor-kappa-B, MLCK-P-MLC: myosin light-chain kinase-phosphorylated-myson ligh chain, VDR: vitamin D receptor, MUC2: mucoprotein2, RELMβ: resistin-like molecule β, DP1: D prostanoid receptor 1, TFF1/2/3: trefoil factor peptides 1/2/3, MUC13: mucoprotein13, PI3K/Akt/mTOR: phosphatidylinositol 3-kinase/Akt/mammalian target of rapamycin, Reg IIIγ: regenerating family member gamma, HIF-1α: hypoxia-inducible factor-1α, IBS-SSS: IBS symptom severity scale, IBS-QOL: IBS quality of life scale, CD36: Cluster Determinant 36, NPC1L1: Niemann-Pick C1-Like1, SR-B1: scavenger receptor, class B type 1, IL-1β: interleukin-1β, IL-6: interleukin-6, TNF-α: tumor necrosis factor-α, PPARγ: peroxisome proliferator-activated receptor γ, IL-17: interleukin-17, TLR4: Toll-like receptor4, CD14: cluster of differentiation 14, ROS: reactive oxygen species, SCFAs: short-chain fatty acids, DAO: diamine oxidase, ↑: increase, ↓: decrease.
